# Supplementary material for: Origin of breath isoprene in humans is revealed via multi-omic investigations
Source: Commun Biol. 2023 Sep 30;6:999. doi: 10.1038/s42003-023-05384-y (PMC10542801; doi:10.1038/s42003-023-05384-y)
Supplement: Supplementary file 2 — Supplementary Information [file 42003_2023_5384_MOESM2_ESM.pdf]

Origin of breath isoprene in humans is revealed via multi-omic investigations

Pritam Sukul[1]*†, Anna Richter[2]†, Christian Junghanss[2], Jochen K Schubert[1], Wolfram Miekisch[1]

[1]Rostock Medical Breath Research Analytics and Technologies (ROMBAT), Dept. of Anesthesiology, Intensive Medicine and Pain Therapy, University Medicine Rostock, Schillingallee 35, 18057 Rostock, Germany.

[2]Department of Medicine, Clinic III – Hematology, Oncology, Palliative Medicine, Rostock University Medical Center, Ernst-Heydemann-Strasse 6, 18057 Rostock, Germany.

*e-mail: pritam.sukul@uni-rostock.de

† These authors contributed equally to this work.

Supplementary Information

Age vs.<br>Breath isoprene	Correlation coefficient	p-value
Overall (n = 2000)	0.134	<0.001
All without<br>isoprene aberrations (n = 1931)	0.159	<0.001
Age <20 years (n = 345)	0.520	<0.001
Age 20 - 60 years<br>(Isoprene normal, n = 1318)	0.069	0.012
Age 20 - 60 years<br>(Isoprene deficient, n = 64)	- 0.135	0.286
Age >60 years (n = 268)	- 0.413	<0.001

Supplementary Table 1. Correlations between biological age (in years) and exhaled isoprene concentrations (ppbV). Spearman correlation test was applied due to the non-parametric (as per Shapiro-Wilk test for normality) distributions of data. Number of observations (n), correlation coefficient and corresponding p-value are provided.

Kruskal-Wallis One Way Analysis of Variance on Ranks					
Data source: *Quantified isoprene data_2000.JNB*					
**Normality Test (Kolmogorov-Smirnov):**	Failed	(p<0.05)			
**Groups**	**N**	**Missing**	**Median**	**25%**	**75%**
Sub.Aged<20 Y	345	0	76.03	43.67	113.18
Iso.Nor_Adults	1318	0	137.63	103.55	186.83
Iso.Def_Adults	64	0	38.28	27.26	43.27
Iso.Abs_Adult	5	0	-12.30	-12.95	-11.46
Sub.Aged>60Y	268	0	100.34	67.43	140.02
Iso_Room air	2000	0	2.724	1.40	4.63
H = 3106.43 with 5 degrees of freedom. (p = <0.001)					
The differences in the median values among the treatment groups are greater than would be expected by chance; there is a statistically significant difference (p = <0.001)					
To isolate the group or groups that differ from the others use a multiple comparison procedure.					
**Multiple Comparisons versus Control Group (Dunn's Method):**					
**Comparisons**	**Diff of Ranks**	**Q**	**P**	**p≤0.005**	
Iso.Abs_Adult vs Iso.Nor_Adult	3186.53	6.15	<0.001	Yes	
Iso_Room air vs Iso.Nor_Adults	2180.45	53.21	<0.001	Yes	
Iso.Def_Adult vs Iso.Nor_Adult	1075.57	7.27	<0.001	Yes	
Sub.Aged<20Y vs Iso.Nor_Adults	601.70	8.61	<0.001	Yes	
Sub.Aged>60Y vs Iso.Nor_Adults	383.79	4.96	<0.001	Yes	
**The multiple comparisons on ranks do not include an adjustment for ties.**					

Supplementary Table 2. Comparison of differences and statistical significance in isoprene concentrations between groups. Statistical significances were tested by means of Kruskal-Wallis one-way ANOVA on ranks (Kolmogorov-Smirnov test for normality followed by the multiple comparisons via post-hoc Dunn's method at p-value ≤0.005). Statistically significant differences with respect to 'isoprene normal adults (Iso.Nor_Adult)' are presented with corresponding difference of ranks. Detailed data on each group size (N), group median, difference in ranks and corresponding p-values are presented.

**Population**	**Frequency homozygotes (%)**	**Frequency heterozygotes (%)**
Latino	0.2033	5.6663
East Asian	0.0000	0.0000
South Asian	0.0415	1.5961
European	0.2265	6.7257
African	0.1208	4.6192
Ashkenazi Jewish	0.0865	4.0634
European-American	0.3372	7.3140
African-American	0.1362	4.6073

Supplementary Table 3. *IDI2* variant frequency in other ethnicities.
