# Supplementary material for: Origin of breath isoprene in humans is revealed via multi-omic investigations
Source: Commun Biol. 2023 Sep 30;6:999. doi: 10.1038/s42003-023-05384-y (PMC10542801; doi:10.1038/s42003-023-05384-y)
Supplement: Supplementary file 3 — Description of Supplementary Materials [file 42003_2023_5384_MOESM3_ESM.docx]

**Description of Additional Supplementary Files**

**File name:** Supplementary Data 1

**Description:** Exhaled alveolar (room air deducted) isoprene concentrations source data behind main Figure 1.
