# Supplementary material for: Origin of breath isoprene in humans is revealed via multi-omic investigations
Source: Commun Biol. 2023 Sep 30;6:999. doi: 10.1038/s42003-023-05384-y (PMC10542801; doi:10.1038/s42003-023-05384-y)
Supplement: Supplementary file 5 — Reporting Summary [file 42003_2023_5384_MOESM5_ESM.pdf]

nature portfolio

Corresponding author(s): Pritam Sukul

Last updated by author(s): Sep 12, 2023

Reporting Summary

Nature Portfolio wishes to improve the reproducibility of the work that we publish. This form provides structure for consistency and transparency in reporting. For further information on Nature Portfolio policies, see our Editorial Policies and the Editorial Policy Checklist.

Statistics

For all statistical analyses, confirm that the following items are present in the figure legend, table legend, main text, or Methods section.

n/a	Confirmed	
☐	☒	The exact sample size (*n*) for each experimental group/condition, given as a discrete number and unit of measurement
☐	☒	A statement on whether measurements were taken from distinct samples or whether the same sample was measured repeatedly
☐	☒	The statistical test(s) used AND whether they are one- or two-sided<br>*Only common tests should be described solely by name; describe more complex techniques in the Methods section.*
☐	☒	A description of all covariates tested
☐	☒	A description of any assumptions or corrections, such as tests of normality and adjustment for multiple comparisons
☐	☒	A full description of the statistical parameters including central tendency (e.g. means) or other basic estimates (e.g. regression coefficient) AND variation (e.g. standard deviation) or associated estimates of uncertainty (e.g. confidence intervals)
☒	☐	For null hypothesis testing, the test statistic (e.g. *F*, *t*, *r*) with confidence intervals, effect sizes, degrees of freedom and *P* value noted<br>*Give P values as exact values whenever suitable.*
☒	☐	For Bayesian analysis, information on the choice of priors and Markov chain Monte Carlo settings
☒	☐	For hierarchical and complex designs, identification of the appropriate level for tests and full reporting of outcomes
☒	☐	Estimates of effect sizes (e.g. Cohen's *d*, Pearson's *r*), indicating how they were calculated

Our web collection on statistics for biologists contains articles on many of the points above.

Software and code

Policy information about availability of computer code

Data collection	No software was used.
Data analysis	No software was used.

For manuscripts utilizing custom algorithms or software that are central to the research but not yet described in published literature, software must be made available to editors and reviewers. We strongly encourage code deposition in a community repository (e.g. GitHub). See the Nature Portfolio guidelines for submitting code & software for further information.

Data

Policy information about availability of data

All manuscripts must include a data availability statement. This statement should provide the following information, where applicable:

- Accession codes, unique identifiers, or web links for publicly available datasets
- A description of any restrictions on data availability
- For clinical datasets or third party data, please ensure that the statement adheres to our policy

All disclosable data are available in the main text or the supplementary materials. Raw and processed experimental data (i.e. not under ethical restrictions) is available from the corresponding author upon reasonable request.

Research involving human participants, their data, or biological material

Policy information about studies with human participants or human data. See also policy information about sex, gender (identity/presentation), and sexual orientation and race, ethnicity and racism.

Reporting on sex and gender	Inclusion of both genders was considered in study design. Findings apply to both genders. Gender were determined based on self-reporting (by participant) method.<br>Disaggregated gender data are presented within manuscript. This information has been collected during recruitment in the clinical screening studies. Signed consent has been obtained for anonymously sharing individual-level demographic data from 2000 subjects.<br>Gender based analysis was not necessary for the study aim but gender distribution is demonstrated within each cohort of this study.
Reporting on race, ethnicity, or other socially relevant groupings	Most of the study participants were of European ethnic origin. We do not expect that the study outcomes are related to ethnicity or race but are most likely transferable to all ethnicities and races.<br>Ethnicities were determined based on self-reporting (by participant) method.<br>Population frequencies of the discovered mutation might differ slightly but this does not interfere with the mechanistic background.
Population characteristics	Study participants were aged between 1-100 years old and included from 15 consecutive clinical breath screening studies conducted for physio-metabolic and clinical assessments.<br>All subjects participated in genetic analysis were healthy adult individuals.
Recruitment	Subjects were recruited from 15 consecutive clinical breath screening studies.
Ethics oversight	Institutional Ethics Committee (IEC), University Medicine Rostock, Rostock, Germany

Note that full information on the approval of the study protocol must also be provided in the manuscript.

Field-specific reporting

Please select the one below that is the best fit for your research. If you are not sure, read the appropriate sections before making your selection.

☒ Life sciences ☐ Behavioural & social sciences ☐ Ecological, evolutionary & environmental sciences

For a reference copy of the document with all sections, see nature.com/documents/nr-reporting-summary-flat.pdf

Life sciences study design

All studies must disclose on these points even when the disclosure is negative.

Sample size	In order to find adults with isoprene aberrations, we re-evaluated the isoprene exhalations in 2000 human subjects (aged between <1 – 100 years) from 15 consecutive clinical breath screening studies by applying real-time mass-spectrometry.
Data exclusions	No data was excluded from analysis.
Replication	Repeatedly measured breathomic data were re-evaluated from 2000 human subjects recruited in 15 consecutive clinical breath screening studies. Whole exome sequencing was not repeated but the lead variant was confirmed via targeted sequencing approach.
Randomization	Randomization was not relevant to our observational screening approach to find natural knock-out cases.
Blinding	Targeted sequencing samples were blinded to the investigator and unblinded only after the analysis of the chromatograms.

Reporting for specific materials, systems and methods

We require information from authors about some types of materials, experimental systems and methods used in many studies. Here, indicate whether each material, system or method listed is relevant to your study. If you are not sure if a list item applies to your research, read the appropriate section before selecting a response.

Materials & experimental systems

n/a	Involved in the study
☒	☐ Antibodies
☒	☐ Eukaryotic cell lines
☒	☐ Palaeontology and archaeology
☒	☐ Animals and other organisms
☒	☐ Clinical data
☒	☐ Dual use research of concern
☒	☐ Plants

Methods

n/a	Involved in the study
☒	☐ ChIP-seq
☒	☐ Flow cytometry
☒	☐ MRI-based neuroimaging
